# Regional specificity of MRI contrast parameter changes in normal ageing revealed by voxel-based quantification (VBQ)

**DOI:** 10.1016/j.neuroimage.2011.01.052

**Published:** 2011-04-15

**Authors:** B. Draganski, J. Ashburner, C. Hutton, F. Kherif, R.S.J. Frackowiak, G. Helms, N. Weiskopf

**Affiliations:** a LREN, Département des Neurosciences Cliniques, CHUV, Université de Lausanne, Lausanne, Switzerland; b Max Planck Institute for Human Cognitive and Brain Sciences, Leipzig, Germany; c Mind Brain Institute, Charité and Humboldt University, Berlin, Germany; d Wellcome Trust Centre for Neuroimaging, UCL Institute of Neurology, UCL, London, UK; e Neuroimaging Laboratory, IRCCS Fondazione Santa Lucia, Rome, Italy; f MR-Research in Neurology and Psychiatry, University Medical Centre, Göttingen, Germany

**Keywords:** Voxel-based morphometry, Voxel-based quantification, Magnetization transfer, Mean diffusivity, Fractional anisotropy, DTI, R1, R2*

## Abstract

Normal ageing is associated with characteristic changes in brain microstructure. Although in vivo neuroimaging captures spatial and temporal patterns of age-related changes of anatomy at the macroscopic scale, our knowledge of the underlying (patho)physiological processes at cellular and molecular levels is still limited. The aim of this study is to explore brain tissue properties in normal ageing using quantitative magnetic resonance imaging (MRI) alongside conventional morphological assessment. Using a whole-brain approach in a cohort of 26 adults, aged 18–85 years, we performed voxel-based morphometric (VBM) analysis and voxel-based quantification (VBQ) of diffusion tensor, magnetization transfer (MT), R1, and R2* relaxation parameters. We found age-related reductions in cortical and subcortical grey matter volume paralleled by changes in fractional anisotropy (FA), mean diffusivity (MD), MT and R2*. The latter were regionally specific depending on their differential sensitivity to microscopic tissue properties. VBQ of white matter revealed distinct anatomical patterns of age-related change in microstructure. Widespread and profound reduction in MT contrasted with local FA decreases paralleled by MD increases. R1 reductions and R2* increases were observed to a smaller extent in overlapping occipito-parietal white matter regions. We interpret our findings, based on current biophysical models, as a fingerprint of age-dependent brain atrophy and underlying microstructural changes in myelin, iron deposits and water. The VBQ approach we present allows for systematic unbiased exploration of the interaction between imaging parameters and extends current methods for detection of neurodegenerative processes in the brain. The demonstrated parameter-specific distribution patterns offer insights into age-related brain structure changes in vivo and provide essential baseline data for studying disease against a background of healthy ageing.

## Research highlights

►High-resolution FLASH-based parameter mapping is suitable for clinical purposes. ►Patterns of age-dependent parameter changes reflect specificity to tissue properties. ►Combining VBM and VBQ offers complementary information about brain architecture.

## Introduction

During the entire life span, the human brain undergoes characteristic structural changes at macroscopic and microscopic levels vaguely described by the term “normal” or “healthy” ageing (Caserta et al., 2009). Age-dependent macroscopic alterations have been extensively studied using high-resolution magnetic resonance imaging (MRI) in vivo combined with computational anatomy methods, thus allowing for the analysis of data in large cohorts (Ashburner et al., 2003). The main findings converge onto a specific pattern of grey matter volume changes in fronto-parietal and subcortical regions (for a review on this topic, see Raz et al., 2007; Thompson and Apostolova, 2007). However, the significance of the morphometric results for understanding the mechanisms of normal ageing is limited since they are not specific with respect to the underlying processes at the tissue/cellular level.

The brain's microanatomy and its relation to age can be studied either post mortem by histological exploration or non-invasively by dedicated quantitative MRI methods. Quantitative MRI reveals the physical properties of water that govern MRI contrast that can be used as surrogate parameters to characterise tissue properties (Tofts, 2003). Here, we study the following major contrast parameters: longitudinal relaxation rate (R1 = 1/T1), effective transverse relaxation rate (R2* = 1/T2*), magnetization transfer saturation (MT), fractional anisotropy (FA) and mean diffusivity (MD). The parameter maps exhibit considerable regional variability; the largest variance is usually due to differences between grey and white matter (GM/WM).

Diffusion tensor imaging (DTI) is an established method to characterise WM micro-architecture (Pierpaoli et al., 1996) and coherence of major fibre tracts (Mori et al., 2008). Established analysis methods like voxel-based and tract-based spatial statistics (TBSS) of DTI parameters are then applied to capture characteristic WM changes (Barrick et al., 2010; Charlton et al., 2006; Giorgio et al., 2010a, 2010b; Kochunov et al., 2007; Mori et al., 2008; Westlye et al., 2010; Zhang et al., 2010). Tensor-derived measures of fractional anisotropy (FA, an index of fibre coherence) and mean diffusivity (MD) show both linear and non-linear dependence on age (Charlton et al., 2006; Giorgio et al., 2010a, 2010b; Salat et al., 2005a; Salat et al., 2005b).

Based on technical developments in recent years, whole-brain high-resolution mapping of R1 and R2* relaxation as well as MT can be performed in clinically feasible acquisition times (Helms et al., 2009; Weiskopf and Helms, 2008). Considering the fact that MT reflects mainly the macromolecular content of tissue it is widely accepted that myelin has a large contribution to measured MT in brain (Bjarnason et al., 2005; Stanisz et al., 1999). Hence, studies characterising WM often combine straightforward mapping of the MT ratio with DTI (Underhill et al., 2009). Histogram peaks are shifted to lower MT ratios in normal ageing (Spilt et al., 2005) with an additional reduction in focal WM hyper-intensities (Scheltens et al., 1995). MT can now be measured without confounding effects of T1- and T2*-weighting of signals, either by fully quantitative (Sled and Pike, 2001) or semi-quantitative approaches (Helms et al., 2008b). According to the common biophysical notion, the rate of longitudinal relaxation (R1) depends mainly on the mobility of water within its microenvironment and the effective transverse relaxation rate (R2*) is sensitive to the iron content of GM (Cherubini et al., 2009a; Duyn et al., 2007; Peran et al., 2009; Peran et al., 2007) but also to WM structure (Li et al., 2009).

Each parameter map, namely FA, MD, R1, R2* and MT saturation, reflects characteristic aspects of tissue micro-architecture and thus has the potential to detect specific changes in normal ageing. So far, the majority of quantitative mapping studies have been restricted to one or two parameters limiting the analysis to predefined ‘regions-of-interest’ (ROI) (Cherubini et al., 2009a; Peran et al., 2009; Underhill et al., 2009). However, ROI analysis is insensitive to changes in other parts of the brain and provides inadequate adjustment for the confounding effects of macro-anatomical volume changes on parameter statistics (Camara et al., 2007).

We hypothesised that normal/healthy ageing induces specific anatomical patterns of changes in GM volume, iron accumulation, myelin loss and water diffusion properties (Barrick et al., 2010; Cherubini et al., 2009a; Giorgio et al., 2010a, 2010b). To test this, we perform voxel-based quantification (VBQ) analyses in the framework of statistical parametric mapping (SPM) for a wide range of (surrogate) parameters – FA, MD, R2*, R1 and MT saturation. Our approach combines a number of recent methodological advances: whole-brain multi-parameter mapping at high resolution, correction of radio-frequency (RF) transmit inhomogeneities using UNICORT (Weiskopf et al., 2011) and diffeomorphic registration using DARTEL (Ashburner, 2007).

## Methods

Twenty-six healthy controls (19 males, age range 18–85 years, mean 52 years) were examined on a 3T whole body MR system (Magnetom TIM Trio, Siemens Healthcare, Erlangen, Germany) using the standard 12-channel RF receive head coil and RF body coil for transmission. Study participants showed no macroscopic brain abnormalities. Care was taken not to include elderly subjects with extended atrophy or with white matter hyper-intensities (WMH) of grade 2 or more by Scheltens' rating scale (Scheltens et al., 1993). Informed written consent was obtained prior to scanning as approved by the local ethics committee.

The whole-brain quantitative MRI protocol comprised (i) multi-parameter mapping based on multi-echo 3D FLASH (fast low angle shot) at 1 mm isotropic resolution (Helms et al., 2009; Weiskopf and Helms, 2008, Weiskopf et al., 2011) as well as (ii) diffusion-weighted imaging (DWI) based on single-shot echo-planar imaging (EPI) of 2.3 mm isotropic resolution (Nagy et al., 2007).

For multi-parameter mapping, three datasets were acquired with predominant T1-, PD-, and MT-weighting imposed by the choice of repetition time (TR) and flip angle (T1: 18.7 ms/20°; PD and MT: 23.7 ms/6°) and by applying an off-resonance Gaussian-shaped RF pulse (4 ms duration, 220° nominal flip angle, 2 kHz frequency offset) prior to non-selective excitation, respectively. Alternating gradient echoes were acquired at six equidistant echo times (TE) between 2.2 ms and 14.7 ms for the T1- and MT-weighted acquisitions with two additional echoes at TE = 17.2 ms and 19.7 ms for the PD-weighted acquisition. A high readout bandwidth BW = 425 Hz/pixel was used to minimise off-resonance artefacts. To speed up data acquisition, GRAPPA parallel imaging with an acceleration factor of 2 was applied in the phase-encoding (anterior-posterior) direction and 6/8 partial Fourier acquisitions in the partition (left-right) direction. The total scanning time for the three FLASH datasets was approximately 20 min. The rationale and detailed design criteria for the protocol are outlined in (Helms et al., 2009; Weiskopf and Helms, 2008). The accuracy and precision of the R1 mapping is assessed in Weiskopf et al. (2011).

DTI was performed with the following parameters: TE = 90 ms, flip angle = 90°, 40 axial slices, matrix 96 ×  96, FOV = 220 mm × 220 mm, slice thickness 2.3 mm, fat saturation, BW = 2003 Hz/pixel, diffusion weighting at a high *b* = 1000 s mm^−2^ along 61 directions and 7 directions at a low *b*-value of 100 s mm^−2^ (Nagy et al., 2007). At 155 ms temporal separation between consecutive slices an interleaved slice acquisition order was chosen to avoid cross talk between adjacent slices.

Since the underlying homogeneity of the static field and thus the R2* maps may systematically depend on the relative orientation of the head, we estimated the individual head position parameters (rotation – range: 14° 12° 20° [*x y z*] and translation – range: 18 mm 13 mm 28 mm [*x y z*]) with respect to standard MNI space. The parameters failed to show a significant correlation with the variable of interest – age (*p* < 0.05).

Data processing was performed in the SPM8 framework (www.fil.ion.ucl.ac.uk/spm) using customised Matlab tools (The MathWorks Inc.; Natick, MA, USA). In brief, the 6 volumes acquired for each weighting were averaged to increase the signal-to-noise-ratio (SNR) (Helms and Dechent 2009). The resulting three averaged volumes were used to calculate the parameter maps of the MT saturation, the apparent longitudinal relaxation rate R1, and the signal amplitude (proportional to PD) as described previously (Helms et al., 2008a; Helms et al., 2008b). The effective transverse relaxation rate R2* was estimated by regression of log signals of the eight PD-weighted multi-echo FLASH volumes (Weiskopf and Helms, 2008). The semi-quantitative MT saturation corresponds to the percentage loss of magnetization imposed by a single MT pulse. It is implicitly corrected for differences in relaxation times and excitation flip angle, thus differing from the commonly used MT ratio, the percentage reduction of the steady state signal (Helms et al., 2010). R1 maps were corrected for bias due to flip angle inhomogeneities applying UNICORT (Weiskopf et al., 2011), which uses the unified segmentation approach (Ashburner and Friston, 2005) to estimate and correct for the RF transmit bias field. For computation of DTI scalar parameters (fractional anisotropy (FA) and mean diffusivity (MD)) we used the Camino freeware (Cook et al., 2006) www.cs.ucl.ac.uk/research/medic/camino).

For VBM analysis, MT maps were classified into GM, WM and CSF using Gaussian mixture model within the unified segmentation approach (Ashburner and Friston, 2005). Images were transformed non-linearly to standard MNI space using the diffeomorphic registration algorithm (DARTEL) implemented in SPM8 (Ashburner, 2007). GM probability maps were subsequently “modulated” by the Jacobian determinants of the deformations to account for local compression and expansion due to linear and non-linear transformation (Good et al., 2001). Finally, GM probability maps were smoothed with an isotropic Gaussian kernel of 6 mm full width at half maximum (FWHM).

In order to achieve optimal parameter assignment to the particular tissue class we implemented a data processing pipeline within the SPM8 framework. The FA maps were affine registered (Maes et al., 1997) to the WM tissue segments, followed by application of the same transformation parameters to the MD maps. The FLASH-based parameter maps (MT, R1 and R2*) and the DTI maps were warped to standard MNI space using the subject-specific diffeomorphic estimates from the DARTEL procedure, but without scaling by the Jacobian determinants (i.e., no “modulation” of parameter values). In order to enhance the specificity for a particular tissue class we additionally performed weighting with the corresponding tissue probability map (GM or WM = *t*) accounting for the partial volume contribution in each voxel. To this end, we implemented a combined weighting/smoothing procedure, which avoids parameter value changes by Gaussian smoothing (*g*) applied in standardised space.

$$\text{signal}=\frac{g*\left(ws\left(\phi \right)\right)}{g*w},$$

where

g* indicates convolution with the Gaussian smoothing kernel,

*w* = weights in standard space, constructed from *w* = |D*ϕ*|*t*(*ϕ*), where

|*Dϕ*| = Jacobian determinants of deformation *ϕ,*

*t*(*ϕ*) = tissue class image warped by *ϕ,*

*s*(*ϕ*) = parameter map (MT, R1, R2*, FA or MD) in standard space warped by *ϕ*.

The procedure transforms the Gaussian smoothing kernel, which is applied in the standardised space, back into subject native space while preserving the weighted average of the parameter value over a region the size of the smoothing kernel. A similar approach has been applied to voxel-based analysis of cortical thickness, diffusion tensor imaging parameters and R1 maps (Hutton et al., 2009; Lee et al., 2009; Taubert et al., 2010; Weiskopf et al., 2011).

For voxel-based statistical analysis of age-dependent regional effects, we used a multiple linear regression model embedded in the General Linear Model framework of SPM8. Age effects were analysed separately for GM and WM sub-space by creating two corresponding design matrices. The only difference between the models was the exclusion of WM volume maps based on MT data due to their a priori low regional specificity. Explicit masking using binary masks of GM and WM ensured inclusion of the same number of voxels in all analyses of each parameter sub-space. The masks were created by averaging over MT-derived GM/WM probability maps using an exclusive logical OR and thresholding at probability value of 0.2. All GM/WM data were included in the same model with regressors for age, gender and total intracranial volume (the sum of grey matter, white matter and CSF volume). Instead of performing analyses on separate design matrices for each parameter we concatenated these using a mass-univariate approach with single block-diagonal design matrix structure. This ensures the same beta parameter estimates as in the separate component analyses. Different variance components (hyper-parameters) were estimated for each block of data separately using REML non-sphericity estimations (Friston et al., 2005). Voxel-based two-tailed *T*-statistics were computed to detect regional effects of age for each parameter map in GM and WM separately. Statistical thresholds were applied at *p* < 0.05 after family-wise error (FWE) correction for multiple comparisons over the whole volume of the GM/WM mask. Trends were assessed by using an auxiliary uncorrected voxel threshold of *p* < 0.001 (Friston et al., 1994). For a descriptive summary of the age-related parameter changes we generated a colour-coded map based on individual *T*-contrasts after binarisation at a threshold of *p* < 0.001 uncorrected for multiple comparisons.

Additionally, we studied the correlation structure between the model and the multi-parametric data by computing eigenvectors in the sub-space of the design matrix defined by *F* contrasts over the age regressors using a whole-brain multivariate linear model (MLM) (Kherif et al., 2002). Linear combinations of parameter maps or eigenvectors that best explain the regressor of interest, i.e.*,* age, were selected based on a statistical threshold of *p* < 0.05.

## Results

**Fig. 1:**
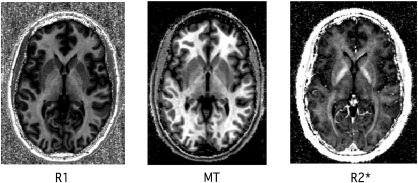
Example of individual parameter data (R1, magnetization transfer – MT and R2*).

**Fig. 2:**
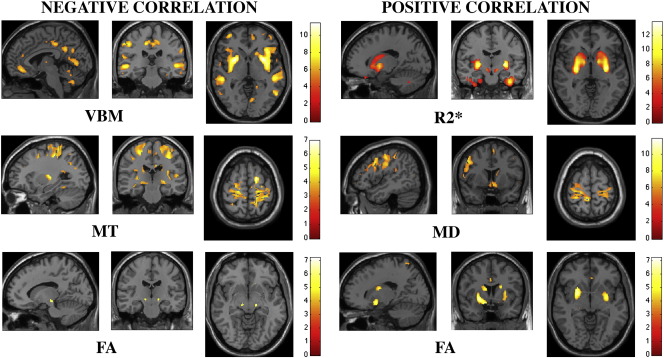
Statistical parametric maps (SPMs) of linear correlation between age and the different parameters based on grey matter voxel-based morphometry (VBM) and voxel-based quantification (VBQ) of R2*, magnetisation transfer (MT), mean diffusivity (MD) and fractional anisotropy (FA). SPMs were thresholded at *p* < 0.001, uncorrected and superimposed for presentation purposes on T1-weighted image.

**Fig. 3:**
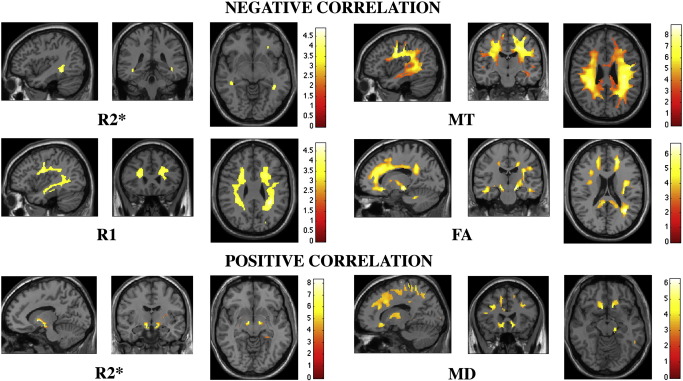
Statistical parametric maps (SPMs) of linear correlation between age and the different parameters based on white matter voxel-based quantification (VBQ) of R1, R2*, magnetisation transfer (MT) fractional anisotropy (FA) and mean diffusivity (MD). SPMs were thresholded at *p* < 0.001, uncorrected and superimposed for presentation purposes on T1-weighted image.

**Fig. 4:**
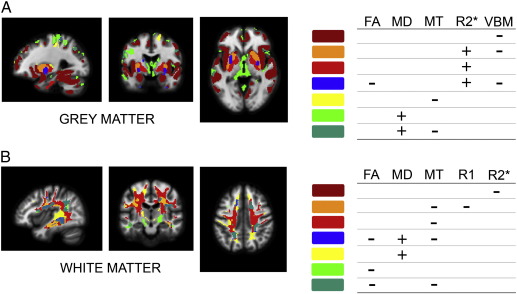
Spatial patterns of age regression T-maps overlap at *p* < 0.001, uncorrected, in grey (A) and white matter (B). Directionality of regression slope (positive or negative correlation) for each parameter is indicated by positive (+) or negative (−) symbol. Overlap between two or more parameters is represented by same colour label. Colour labels of bars correspond to labelling convention on image. FA – fractional anisotropy, MD – mean diffusivity, MT – magnetisation transfer, R1, R2* and VBM (voxel-based morphometry).

**Fig. 5:**
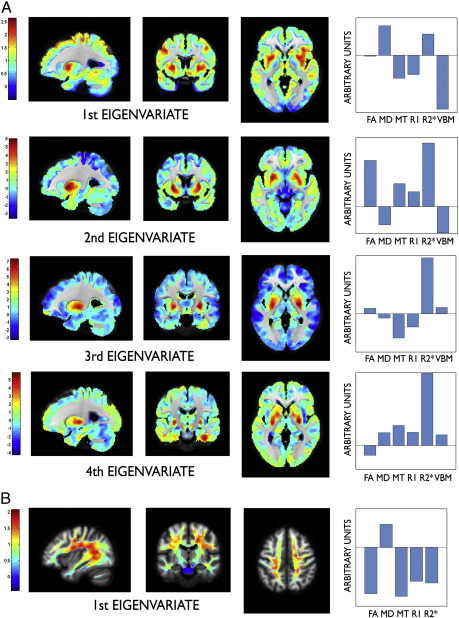
Spatial patterns of statistically significant eigenvariates (*p* < 0.05, Lawley–Hotelling trace) in grey (A) and white matter (B) using multivariate linear model of age regression. 1st and 2nd eigenvariates in grey matter explain 80% of the variance; 1st eigenvariate in white matter explains 86% of the variance. The specific pattern of directionality and magnitude of change in parameter maps is presented in the bar graphs on the right hand side. FA – fractional anisotropy, MD – mean diffusivity, MT – magnetisation transfer, R1, R2* and VBM (voxel-based morphometry, i.e., grey matter volume).

The FLASH-based parameter maps were dominated by the contrast between WM, GM and CSF but showed additional differences between distinct structures within GM or WM (see Fig. 1 for relaxometry parameter maps of a single subject). The observed parameter changes in GM were to a large extent co-localised with regions showing volume alterations, predominantly in subcortical GM (Fig. 2). In WM, however, changes were more widespread comprising frontal, parietal and occipital subcortical areas (Fig. 3). The distinct spatial patterns and their overlap can be appreciated on multi-colour binarised T-maps (Fig. 4) and the results of a multivariate analysis (Fig. 5).

All significant findings of age dependence in the different analyses are summarised in Tables 1–3. Table 1 details the GM volume changes detected by VBM, whereas Tables 2 and 3 detail the parameter changes in GM and WM, respectively.

### VBM of GM volume

We detected symmetric age-related decreases of GM volume in amygdala, striatum, prefrontal, temporal and parietal cortical areas, cerebellum and in the midline structures, cingulate cortex and precuneus (Fig. 2, top left). There were no significant regional volume changes that correlated positively with age.

### VBQ regression analysis of FA

Grey matter: FA increased with age in the putamen bilaterally, the left hippocampus and the middle cingulate gyrus (Fig. 2, bottom right). We also observed trends to positive correlation with age in the right hippocampus, body of caudate bilaterally and rostral cingulate cortex. There was a negative correlation between age and FA in the substantia nigra pars compacta (SNc) bilaterally and a trend towards a negative correlation for the head of the caudate and cerebellar lobule IV bilaterally.

White matter: We demonstrated a significant negative correlation between FA and age in brainstem portions of the cortico-spinal tract and in fronto-striatal, prefrontal, temporo-parietal and cerebellar white mater areas (Fig. 3, mid right). There were no areas where FA increased significantly with age.

### VBQ regression analysis of MD

Grey matter: Ageing was associated with significantly higher bilateral MD in the primary sensorimotor cortex (S1 and M1), body of the caudate, medio-dorsal thalamic nuclei, habenulae, hypothalamus, left operculum, precuneus, and cerebellar vermis (Fig. 2, mid right). There were no findings of MD correlating negatively with age; neither in GM nor in WM.

White matter: There were significant positive correlations between MD and age in white matter areas corresponding to fronto-striatal connections bilaterally and adjacent to the left S1. A corresponding trend at the right S1 did not reach significance. Trends towards positive correlations between age and MD showed up in prefrontal and parietal white matter areas bilaterally (Fig. 3, bottom right).

### VBQ regression analysis of MT

Grey matter: There was a significant negative correlation between MT and age in the left primary motor cortex (M1), bilateral somatosensory cortex (S1), right caudal middle frontal gyrus, cerebellum bilaterally and motor areas within the right putamen (Fig. 2, mid left). The analysis revealed trends for negative correlation between MT and age in right M1, the ventro-lateral thalamic nucleus, dorsal caudate bilaterally and the left dorso-lateral putamen.

White matter: MT decreased significantly with age in widespread white matter regions adjacent to prefrontal, temporo-parietal cortex as well as along the optic radiation (OpR) and in the genu of the corpus callosum (Fig. 3, top right).

### VBQ regression analysis of R1

Grey matter: We did not detect any significant regional changes correlating with age.

White matter: A significant decrease of R1 with age was found in parietal white matter. There was a trend towards a negative correlation in portions of fronto-striatal white matter and inferior longitudinal fasciculi (ILF) bilaterally (Fig. 2, mid left).

### VBQ regression analysis of R2*

Grey matter: We demonstrate significant positive correlations between R2* and age in putamen, pallidum, fusiform gyrus, and substantia nigra bilaterally and in the orbito-frontal cortex (Fig. 2, top right). A trend for positive correlation was observed in the head and body of the caudate bilaterally. There were no significant regional R2* decreases correlated with age.

White matter: An increase of R2* with age was observed in the area of the genu of the internal capsule (IC) bilaterally and we noted an R2* decrease in the right parietal white matter region. A trend for negative correlation was found in the left parietal area and optic radiation bilaterally.

## Discussion

Our results on age-related changes in human brain in vivo demonstrate volume alterations paralleled by specific changes in R1, R2*, MT and DTI parameters with high degree of specificity for distinct tissue type and tissue property. This reflects the parameters’ differential sensitivity for structural aspects of tissue such as fibre coherence, macromolecules, myelin, iron and water content. Quantitative MRI techniques offer through their sensitivity to microstructural tissue properties a unique opportunity for establishing in vivo the link to findings of post mortem histological assessment of brain tissue.

The main technical advantage of our approach is that the entire brain was covered at isotropic spatial resolution, building on state-of-the-art imaging sequences, modelling, and automated unbiased statistical parametric mapping. Previous studies have reported an age-dependence of MT and R2* parameters in predefined ROIs (Cherubini et al., 2009a; Peran et al., 2009; Peran et al., 2007). Distribution patterns of age-related changes as demonstrated separately for both GM and WM have not been reported so far. For this study, an appropriate voxel-based data processing scheme was developed, dubbed voxel-based quantification (VBQ). The VBQ scheme may be readily applied to group comparisons or correlation with clinical parameters. It takes full account of the partial volume information at sub-voxel (or mesoscopic) level provided by VBM. To summarise the age-related parameter changes we generated a statistical map based on a conjunction analysis of parameter-specific differential contrasts and explored the covariance of the parameter maps using a multivariate linear model projecting the data in the sub-space of the design matrix over the age regressors (Friston et al., 2005; Kherif et al., 2002). We demonstrate the similarity between spatial patterns derived from univariate and multivariate analyses (Figs. 4 and 5). A major challenge for future studies will be to estimate interactions and causal relationships among parameters.

We took advantage of a more reliable characterisation of subcortical structures based on automated tissue classification using MT maps with exquisite contrast between GM and WM in deep brain regions (Helms et al., 2009). Specifically for WM, the parameter maps demonstrate widespread tissue heterogeneity, corroborating findings based on T2*-weighted gradient-echo MRI (Duyn et al., 2007; Li et al., 2009) and quantitative MT imaging (Yarnykh and Yuan, 2004). These are not visible on T1w MRI where WM appeared quite homogeneous.

### Volume changes

Our results showed age-related grey matter volume decreases mainly in fronto-parietal, temporal and subcortical regions in line with previous computational anatomy studies (Giorgio et al., 2010a, 2010b; Good et al., 2001; Thompson and Apostolova, 2007, Raz et al., 2007, Westlye et al., 2010). Despite the widespread use of VBM for studying age-related changes, to our knowledge, there are no conclusive cross-correlation validation studies with defined experimental models of (patho)physiological processes underlying the morphometric findings. Basic mechanisms associated with normal ageing include a decrease in neuron number, loss of intracortical myelin, axons and dendritic spines, microglial activation, pathological iron storage, oxidative stress and compromised DNA repair affecting intracellular proteins/cellular skeleton (Brown, 2009; Marner et al., 2003; Pakkenberg et al., 2003; Yankner et al., 2008). The demonstrated age-dependent cortical volume loss is consistent with quantitative stereological analyses on post mortem specimens showing a 10% reduction in numbers of neocortical neurons with relative preservation of neuronal size and synaptic density (Pakkenberg et al., 2003). The demonstrated anterior cerebellar lobe volume changes corroborate volumetric results (Kennedy and Raz, 2005; Raz et al., 2005) and histological findings showing 40% reduction of the total number of granule and Purkinje cells and a 28% loss of anterior cerebellar lobe cortical volume with ageing (Andersen et al., 2003).

### Interpretation of parameter changes in view of biophysical tissue models

In the following section we discuss our results in the light of histological and computational anatomy findings acknowledging existing biophysical tissue models. Current concepts of WM degeneration follow the notion of an anterior to posterior gradient of myelin breakdown and the “last in, first out” hypothesis, suggesting that brain regions that are last to develop are the first to atrophy (Bartzokis et al., 2004). An alternative hypothesis suggests age-related specific vulnerability of early-myelinating, high-workload pathways due to deficient maintenance (Bartzokis, 2009).

DTI: We detected symmetric bilateral FA decreases in WM, comprising prefrontal, parietal regions and the diencephalic portion of the cortico spinal tract (CST). These findings are in agreement with recent longitudinal MRI results suggesting a similar age-related pattern of FA changes, with no evidence for preferential frontal FA involvement (Barrick et al., 2010). FA decreases were paralleled by MD increases in prefrontal and parietal WM, corroborating results from previous DTI studies (Burzynska et al., 2010; Camara et al., 2007; Kochunov et al., 2007; Salat et al., 2005a; Salat et al., 2005b, Giorgio et al., 2010a, 2010b; Wu et al., 2011). In view of the possible physical and physiological confounds of studies correlating post mortem DTI and histology (D'Arceuil and de Crespigny, 2007; Schmierer et al., 2007) our interpretation of age-related FA and MD changes in WM builds on a recent study comparing in vivo DTI measures with histo-pathological findings (Concha et al., 2010). The authors demonstrated a strong positive correlation between FA and axon diameter (cumulative axonal membrane circumference), which further influenced the magnitude of perpendicular diffusion. Further, FA showed only a trend towards positive correlation with axonal density, negative correlation with myelin thickness and no correlation with myelin and extra-axonal fractions. Although these results were drawn from patients with temporal lobe epilepsy and generalisations should be met with caution, we acknowledge the reduction of axonal diameter, axonal density and increase in proportion of dysfunctional thick myelin sheaths as the most significant factors driving age-related, concomitant FA and MD changes in WM.

In view of these findings, the age-related decrease of FA in the CST cannot be explained by reduction of small myelinated fibres as demonstrated using quantitative histological analysis (Terao et al., 1994). Our biophysical interpretation builds on the notion that FA decreases in the CST which are not accompanied by MD changes correspond to loss of fibre coherence without or with small amount of tissue loss (Zhang et al., 2010). The demonstrated preferential involvement of CST regions compared to other regions with a high degree of fibre coherence, such as the optic radiation or corpus callosum, is in line with both the “last in, first out” hypothesis as well as with the model predicting selective age-related degeneration of areas with high functional demands and increased need for maintenance (Bartzokis, 2009). Both alternative hypotheses accommodate histological findings reporting 30–45% age-related reduction of myelinated fibers and our findings with concomitant changes of FA and MD in the subcortical WM (Marner et al., 2003; Tang et al., 1997).

The majority of previous DTI studies restricted the analysis to brain regions with sufficient coherence (e.g., FA > 0.2), thus excluding major portions of the cortical and subcortical GM. We analysed DTI parameters also in GM considering the fact that the VBQ approach does not require an arbitrary threshold to be set. Cortical and subcortical GM structures show both concordant and discordant age-dependent changes in FA and MD. An age-related FA increase without corresponding change in MD (as seen in the basal ganglia) may represent altered directional organisation of grey matter. Alternatively, it may also be explained in terms of signal physics by T2-related signal losses in the presence of increasing iron content (as indicated by increased R2*) predominantly affecting more freely diffusing water molecules. Similarly, FA increases in putamen have been reported in a recent study using ROI based measurements (Zhang et al., 2010). Further, our MD findings indicating increased water diffusivity in hippocampus and primary somatosensory cortex can be interpreted as reflecting tissue density decreases (e.g., loss of dendritic arborisations).

MT: Our observation of widespread MT decreases in subcortical WM areas is in line with histopathological findings demonstrating age-related WM changes caused by dysfunctional repair/production in small diameter myelinated fibres (Marner et al., 2003; Pakkenberg and Gundersen, 1997; Peters, 2002; Tang et al., 1997). MT in WM can behave differently from DTI parameters during ageing, representing different physical mechanisms as described in a recent study (Underhill et al., 2009). The apparent discrepancy between MT and DTI parameters in the mesencephalic portion of the CST may be explained by considering that the strong MT effect on water trapped between the myelin layers (MacKay et al., 1994) needs to be transferred to bulk water for full effect (Stanisz et al., 1999). Considering the fact that the CST consists of a mixture of axons with different sizes, water from axons with thinner myelin sheaths will exchange faster than that from thicker myelin sheaths. According to this concept, age-related alterations of fibre coherence will impact more strongly on FA than on MT. Also, the loss of thin axons will contribute disproportionately to MT whereas FA will tend to be affected only marginally. In contrast to a previous report we failed to detect age-dependent MT differences at the ventricular boundary (Camara et al., 2007). This can be explained by the higher resolution of the MT maps, the excellent performance of the diffeomorphic registration minimising registration inaccuracies and by the exclusion of subjects with periventricular WMH with altered MT (Fazekas et al., 2005).

We detect a characteristic pattern of age-dependent MT reduction localised predominantly in cortical and subcortical GM structures of the “motor” cortico-basal ganglia-cerebellar circuit. Similar to WM changes, we suggest that MT in GM may predominantly reflect the decline of a “structural” component. MT saturation maps show a high contrast between GM and CSF or WM, as well as improved contrast within GM (Gringel et al., 2009). The supposition here is that MT reflects different axonal content as supported by direct comparison to DTI parameters (Underhill et al., 2009). Supporting the general notion of MT reduction reflecting a decrease of “structural” components the observed MT decline can be ascribed to changes in myelin content as seen in cortical regions of the “motor” circuit. The regional specificity of MT grey matter changes with predominant involvement of the sensorimotor cortex can be interpreted by the sensitivity of MT to the age-related density reduction of small myelinated fibres in the CST described previously using quantitative histological methods (Terao et al., 1994). An alternative explanation accommodating involvement of sensorimotor rather than other functional areas (e.g., primary visual areas) builds on the idea of specific age-dependent vulnerability of phylogenetically recent, high-workload areas related to fine motility of the hands, bipedal locomotion and posture (Ghika, 2008).

R1: R1 maps revealed bilateral changes in WM mainly clustered along prefrontal and parietal subcortical WM structures. Both the spatial extent and the effect size of R1 WM changes were smaller compared to MT, although both maps show a high degree of correlation due to MT contributions to the observed R1 (Helms and Hagberg, 2009). R1 is predominantly influenced by water content and less so by iron content (Gelman et al., 2001). However, R1 seems to be less sensitive than MT or R2*, respectively, in monitoring these aspects of ageing. This is in line with converging evidence from quantitative studies for widespread heterogeneity of normal WM in terms of water content and T2 (Whittall et al., 1997), quantitative MT (Yarnykh and Yuan, 2004), MT and DTI (Underhill et al., 2009) or R2* (Li et al., 2009), which are marginally represented in the T1 relaxation rate R1 (Helms et al., 2008a). From a methodological point of view, the lower sensitivity of R1 may be due to the influence of flip angle bias on R1 (Helms et al., 2008a), the correction of which required additional processing (Weiskopf et al., 2011).

R2*: We demonstrate differential anatomical patterns corresponding to positive and negative correlations between R2* and age in WM. R2* increases with age in the diencephalic portions of CST, whereas trends for R2* decreases with age were detected in the optic radiation and in parietal WM areas. The concordance with MT and DTI parameter changes in the diencephalic portions of CST suggests predominant “structural” loss as opposed to long-ranging B0 field inhomogeneities radiating from adjacent midbrain structures with increased iron content. Here, non-linear B0 inhomogeneities may be responsible for the R2* increase, since 3D R2*-mapping by short TE trains, in contrast to 2D techniques, is hardly affected by local B0 gradients (Helms and Dechent, 2010), non-linear B0 inhomogeneities may be responsible for the R2* effects in the CST. Considering recent evidence for R2* depending on the orientation of WM structure with respect to the main magnetic field (Cherubini et al., 2009b) we consistently positioned all subjects and found no post hoc evidence for any correlation between individual head position parameters and the variable of interest – age.

Bilateral R2* increases in basal ganglia and STG as well as in the left operculum correlated with age. Post mortem histological studies (Hallgren and Sourander, 1958) and MR studies are consistent with age-dependent increased iron content (Bartzokis et al., 2007; Cherubini et al., 2009a; Peran et al., 2009). Due to the fact that the MT saturation remains almost unaffected by underlying alterations of R1 or R2*, an absence or an increase of MT may help identify whether the cause of R2* increases is an increase in intracellular iron or in axonal density. Iron changes and myelination may be seen as parts of the same process as suggested by a recent susceptibility mapping study exploring the cortical myeloarchitecture (Fukunaga et al., 2010). The described regionally specific co-localisation of myelin and iron in both WM and GM, points towards the role of iron in cholesterol and lipid synthesis, in myelination maintenance and repair in adult mammals (Cheepsunthorn et al., 1998). Additionally, the findings confirm the importance of brain iron in the form of ferritin as a dominant contributor to R2* contrast with intracortical variation explained by the high iron content of intracortical fibres.

### Methodological considerations

The high-resolution FLASH-based parameter mapping is suited also for clinical purposes. We achieve whole-brain isotropic voxel size of 1 mm in only 19 min acquisition time, which is comparable with the acquisition time of 13 min for a single T1w high-quality image for VBM (Deichmann et al., 2004). The estimation of parameter maps from the raw data (Helms et al., 2008a; Helms et al., 2008b; Weiskopf et al., 2011) and VBQ evaluation by statistical models is integrated within the SPM framework. The computed parameter maps have an isotropic resolution of 1 mm, which, after optimal diffeomorphic registration into standardised space, provides sufficient anatomical detail for further statistical analysis. DARTEL registration minimises bias caused by misregistration of areas particularly close to tissue boundaries.

We did not attempt to explicitly model a non-linear dependency of parameters on age or determine an end-point for the parameters used mainly because of the limited number of study participants. Since individual ageing trajectories increase variability towards the end of the life span, much larger cohorts are required for reliable non-linear modelling. To identify a regional pattern, linear regression is the most conservative model with the fewest assumptions about time courses. However, differences in the time course of age-dependent changes between parameters may have obscured changes and co-localization of regional alterations.

Another limitation is intrinsic to the current usage of DTI voxel-based analysis which did not include an adjustment for the presence or absence of crossing fibres, thus potentially leading to false-positive/-negative inferences. DTI results may also be improved further by explicit correction of susceptibility-related distortions (Andersson et al., 2003; Gallichan et al., 2010). However, the striking similarity between our findings and tract-based analysis of age-dependent FA changes (Barrick et al., 2010; Hugenschmidt et al., 2008) provide face validity for our results.

The patterns of parameter changes were assessed using standard mass univariate statistical analysis (Friston et al., 2002). It is a powerful approach, since it allows unbiased whole-brain analysis of large datasets and reliably controls the statistical family-wise error. However, the multi-parameter data does lend itself well to multivariate statistical approaches, since several outcome parameters are available for each voxel. In particular, a comparison between Figs. 4 and 5 suggests that a multivariate analysis results in a spatially similar pattern of age-related parameter changes, i.e., regionally specific “fingerprints.” This can be appreciated in the example of age-dependent changes on pallidum and putamen. These are distinguishable both in the univariate (red vs. orange colour label) and multivariate (3rd and 4th eigenvariate vs. 1st eigenvariate) analyses. Further elaboration of multivariate statistical methods could be a principled way to determine the directionality of interactions between parameter maps. This could help to establish the VBQ approach for automated machine learning-based classification of ageing and disease as well as for prediction of demographic and/or clinical outcome variables.

## Conclusions

We developed a comprehensive approach combining morphometric and quantitative MRI approaches, thus offering complementary information regarding brain architecture in normal ageing. While VBM reflects mesoscopic GM volume changes and FA is associated with axonal coherence, MT seems particularly sensitive to the content of structural material, including myelin, but not to local anisotropy. Similar to MT, the observed pattern suggests that R1 may be predominantly affected by “structural” tissue components, hence primarily demonstrating changes in myelin content. Finally, R2* age-related changes in WM can be due to accumulation of iron deposits in the process of remyelination or due to cellular/structural loss. The spatial patterns of changes observed for the distinct parameters and their anatomical specificity might be explained by their differential specificity to underlying tissue properties. Despite converging evidence further comparative post mortem studies with emphasis on histological correlation are clearly necessary to affirm the interpretation of multi-parameter data in terms of underlying (patho-)physiological processes and tissue changes. We conclude that voxel-based quantification (VBQ) of parameter maps adds to established VBM analyses through sensitivity to age-related changes at levels of the macroscopic morphology and of tissue microstructure.

### Statistical analysis

Conducted by the first author (B.D.).

### Disclosure

The authors report no conflicts of interest.

## Figures and Tables

**Table 1:** Summary of VBM results (*p*_FWE_ < 0.05). Coordinates [*x y z*] refer to the Montreal Neurological Institute (MNI) standard stereotactic space.

VBM analysis	Region	Left hemisphere coordinates(mm)			*t* score	*z* score	Right hemisphere coordinates(mm)			*t* score	*z* score
*x*	*y*	*z*	*x*	*y*	*z*
GM											
Age-negative correlation	insula	−40	26	0	6.1	5.7	38	21	0	9.2	Inf
cerebellum	−36	−58	−32	7.4	6.7	39	−58	−32	9.7	Inf
DLPFC	−39	11	29	8.5	7.6	42	9	36	7.9	7
cuneus	−9	−63	13	6.9	6.4	18	−60	19	8.5	7.6
IPC	−59	−33	43	7.8	7	27	8	0	11.4	Inf
precuneus	−8	−54	33	6.1	5.7	6	−51	31	7.3	6.7
caudate	−17	8	16	7.2	6.6	17	14	14	7.3	6.7
putamen	−27	6	0	11.1	Inf	27	8	0	11.4	Inf
STG	−54	−30	−5	9.1	Inf	51	−29	−3	8.4	7.6
frontal pole	−36	54	0	7.5	6.9	30	57	5	7.1	6.5
M1	−45	−15	38	7.5	6.9	45	−12	38	7.7	7
rACC	−5	42	−14	7.4	6.8	4	39	−12	7.2	6.6
middle cingulate	−12	−27	42	8.2	7.3	12	−22	43	7.8	7.1
amygdala	−27	−2	−18	7.5	6.9	27	−2	−21	8.9	7.9
fusiform gyrus	−23	−67	−11	7.2	6.6	30	−47	−9	9.6	Inf

GM – grey matter; DLPFC – dorso-lateral prefrontal cortex; IPC – inferior parietal cortex; rACC – rostral anterior cingulate cortex; M1 – primary motor cortex; STG – superior temporal gyrus.

**Table 2:** Summary of VBQ results within grey matter (*p*_FWE_ < 0.05), uncorrected results (*p* < 0.001) are highlighted with an asterisk. Coordinates [*x y z*] refer to the Montreal Neurological Institute (MNI) standard stereotactic space.

VBQ analysis	Region	Left hemisphere coordinates (mm)			*t* score	*z* score	Right hemisphere coordinates (mm)			*t* score	*z* score
*x*	*y*	*z*	*x*	*y*	*z*
FA GM											
Age-positive correlation	middle cingulate						15	−16	43	5	4.8
hipp	−9	−39	3	6	5.7	12	−36	3	4.7*	4.5*
putamen	−24	3	−8	6.3	5.9	30	−4	−9	5.2	4.9
caudate	−17	−1	15	4.8*	4.6*	21	2	18	4.5*	4.4*
rACC	0	29	−14	4*	3.8*					
FA GM											
Age-negative correlation	caudate	−8	21	−5	4.2*	4.1*	10	23	−6	6.1	5.7
cerebellum V	−11	−48	−23	5.5	5.2	12	−46	−24	5.1	4.8
SNc	−14	−19	−9	5.9	5.6	15	−19	−9	6.2	5.8
MD GM											
Age-positive correlation	S1	−27	−38	66	7.5	6.8	29	−35	66	6.5	6.1
thalamus habenula	−3	−25	0	7.9	7.1	3	−24	−3	6.9	6.4
thalamus MD	−9	−3	9	6.5	6.1	10	−6	12	5.7	5.4
M1	−30	−27	58	8.3	7.4	27	−26	66	6.7	6.2
operculum	−50	12	30	8.8	7.8					
hypothalamus	−3	5	−14	6.6	6.2	6	5	−14	6.8	6.3
caudate	−8	14	4	5.7	5.4	10	17	6	7.4	6.7
precuneus						11	−69	27	7.3	6.6
cerebellum vermis	−3	−76	−27	7.1	6.5	4	−64	−9	11.8	Inf
MT GM											
Age-negative correlation	S1	−21	−27	53	6	5.6	31	−28	48		
caudal middle frontal						16	0	53		
caudate	−17	−11	17	4.5*	4.4*	13	−6	16	7	6.4
M1	−17	−27	67	5.1	4.8	27	−24	66	6.9	6.4
putamen	−31	−23	3	4.2*	4.1*	28	−21	4	4.3*	4.2*
thalamus VL						16	−15	3	4.1*	4*
caudate	−15	−14	17	5.4*	4.3*	17	−12	19	4.7*	3.8*
cerebellum	−14	−56	−32	5.1	4.8	17	−55	−39	5.2	5
R2* GM											
Age-positive correlation	SNc	−11	−13	−12	5.7	5.4	12	−12	−11	6	5.6
OFC	−2	30	−27	5.6	5.3					
putamen	−27	−12	−2	12	Inf	28	−10	0	13.8	Inf
pallidum	−14	2	−5	8.7	7.7	13	2	−5	9.1	Inf
fusiform	−47	−16	−29	7.7	7	37	−13	−36	9.4	Inf
caudate	−18	−2	15	4.8*	4.6*	18	5	14	4.5*	4.3*

GM – grey matter; FA – fractional anisotropy; MD – mean diffusivity; MT – magnetization transfer; DLPFC – dorso-lateral prefrontal cortex; OFC – orbito-frontal cortex; rACC – rostral anterior cingulate cortex; M1 – primary motor cortex; S1 – primary somatosensory cortex; thalamus MD – thalamic medio-dorsal nucleus; thalamus VL – thalamic ventro-lateral nucleus; SNc – substantia nigra pars compacta; hipp – hippocampus.

**Table 3:** Summary of VBQ results within white matter (*p*_FWE_ < 0.05), uncorrected results (*p* < 0.001) are highlighted with an asterisk. Coordinates [*x y z*] refer to the Montreal Neurological Institute (MNI) standard stereotactic space.

VBQ analysis	Region	Left hemisphere coordinates(mm)			*t* score	*z* score	Right hemisphere coordinates(mm)			*t* score	*z* score
*x*	*y*	*z*	*x*	*y*	*z*
FA WM											
Age-negative correlation	ILF	−47	−18	−18	5.1	4.8	45	−21	−15	4.6	4.4
PLIC	−12	−8	−5	3.5*	3.2*	26	−18	2	4.8	4.5
fronto-striatal	−20	32	−11	5.6	5.2	19	24	−12	5.7	5.3
CST	−14	−19	−12	5.4	5.1	15	−21	−12	6.5	6
prefrontal	−18	44	7	5.6	5.2	19	38	21	6	5.5
parietal	−17	−49	24	5.3	5	34	−57	18	6.7	6.1
cerebellum	−14	−45	−29	5.8	5.4	16	−42	−30	8.2	5.5
MD WM											
Age-positive correlation	S1	−33	−33	57	4.6	4.4	26	−33	62	4.4*	4.2*
fronto-striatal	−9	17	−15	4.8	4.5	9	21	−7	6	5.5
prefrontal	−30	23	27	4.4*	4.2*	32	23	30	4*	3.9*
parietal	−41	−60	33	4.1*	4*	20	−57	47	4.2*	4*
MT WM											
Age-negative correlation	prefrontal	−25	20	24	5.1	4.8	29	29	26	5.1	4.8
fronto-striatal	−18	35	−9	4.4*	4.2*	17	44	−11	6	5.6
M1	−24	−18	54	7.5	6.4	24	−17	59	8.7	7.6
S1	−21	−30	42	7.4	6.2	20	−42	54	7.1	5.9
OpR	−30	−68	6	7.2	6	39	−48	2	6.7	5.7
ILF	−36	−45	−8	5.5	5.2	40	−32	−6	5.2	4.9
Genu CC	−8	27	0	4.6	4.4	12	32	6	4.9	4.7
R1 WM											
Age-negative correlation	prefrontal	−25	3	33	4.3*	4.1*	23	5	32	4.1*	3.9*
fronto-striatal	−28	24	15	4.4*	4.2*	10	27	10	5.1	4.1
parietal	−35	−49	7	4.5	4.3	33	−56	14	4.8	4.6
ILF	−44	−18	−17	3.8*	3.7*	42	−22	−18	4.9	4.6
R2* WM											
Age-positive correlation	IC	−9	−1	−3	6	4.6	11	0	−3	6.7	4.9
R2* WM											
Age-negative correlation	OpR	−35	−57	2	3.9*	3.4*	35	−57	2	4.3*	3.6*
parietal	−39	−40	30	4*	3.5*	27	−43	31	4.3	3.7

WM – white matter; FA – fractional anisotropy; MD – mean diffusivity; MT – magnetization transfer; ILF – inferior longitudinal fascicle; PLIC – posterior limb of the internal capsule; IC – internal capsule; CST – cortico-spinal tracts; M1 – primary motor cortex; S1 – primary somatosensory cortex; OpR – optic radiation; CC – corpus callosum.
